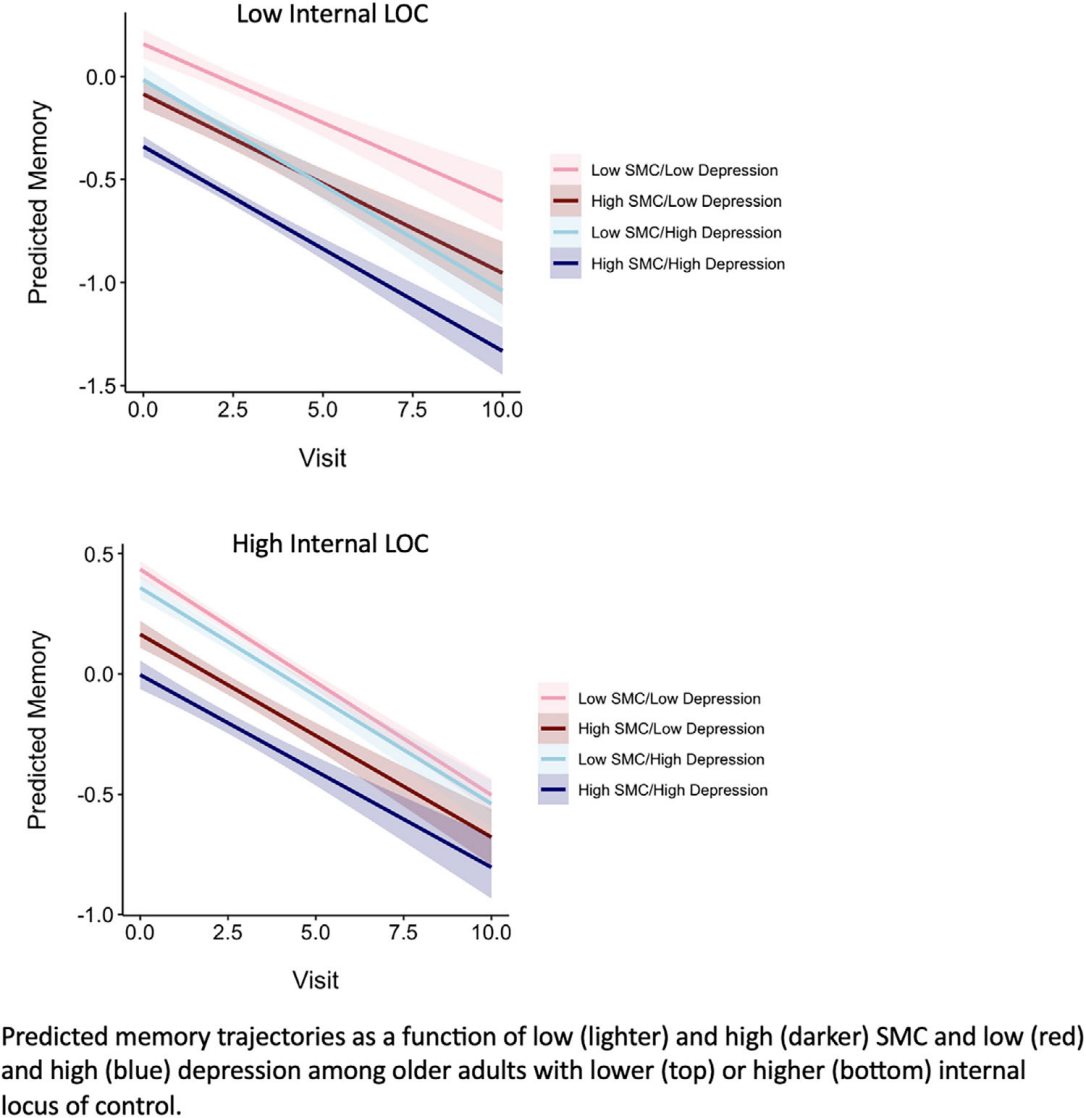# Subjective and objective memory among older adults: Examining the intersecting role of depression and locus of control in the ACTIVE study

**DOI:** 10.1002/alz.093436

**Published:** 2025-01-03

**Authors:** Alexandra J. Weigand, Alexandra L. Clark, Mary Ellen Garcia, Jeanine M Parisi, Laura B. Zahodne, Ian M McDonough, Michael Marsiske, Kelsey R. Thomas

**Affiliations:** ^1^ San Diego State University/University of California, San Diego Joint Doctoral Program in Clinical Psychology, San Diego, CA USA; ^2^ University of Texas at Austin, Austin, TX USA; ^3^ VA San Diego Healthcare System, San Diego, CA USA; ^4^ Johns Hopkins Bloomberg School of Public Health, Baltimore, MD USA; ^5^ University of Michigan, Ann Arbor, MI USA; ^6^ Binghamton University, Binghamton, NY USA; ^7^ Florida Alzheimer’s Disease Research Center, Gainesville, FL USA; ^8^ University of California, San Diego, La Jolla, CA USA

## Abstract

**Background:**

Subjective memory concerns (SMC) may be a sensitive marker of future cognitive declines. However, there are multiple factors that can impact the predictive utility of SMC. Prior studies have demonstrated the effect of depression on SMC. Further, perceived control over one’s situation as measured by locus of control (LOC) has been linked to both depression and SMC. Therefore, the current study examined the moderating effect of depressive symptoms on the association between SMC and memory decline and further investigated whether this interaction varied as a function of internal LOC.

**Method:**

This study included 2721 older adults without dementia from the Advanced Cognitive Training for Independent and Vital Elderly (ACTIVE) study. Predictors of interest included SMC, depressive symptom severity, and internal LOC. Multilevel mixed models examined the 4‐way interaction between internal LOC, depressive symptoms, SMC, and time on 10‐year rate of change on an objective memory factor score.

**Result:**

There were significant independent effects of SMC (*t* = ‐2.40, *p* = .02) and depressive symptoms (*t* = ‐3.48, *p* < .001) on 10‐year memory trajectories, but no significant interaction between SMC and depressive symptoms (*t* = 1.45, *p* = .15). However, there was a significant interaction between internal LOC, depressive symptoms, and SMC on memory trajectories (*t* = ‐3.52, *p* < .001). Specifically, in the context of lower internal LOC, depressive symptoms strengthened the association between SMC and objective memory trajectories, whereas depression was not a significant moderator among individuals with higher LOC.

**Conclusion:**

Findings demonstrate the complexities of SMC in predicting memory decline and highlight intersecting individual‐level factors that may influence the predictive utility of SMC among older adults. Specifically, we found that in individuals with lower internal LOC (e.g., less belief they can control their own cognitive aging), the strength of association between SMC and memory decline was strengthened by depressive symptoms. Future research will examine additional interacting factors (e.g., demographics, sociocontextual variables) to help provide a nuanced understanding of when SMC are most prognostically useful.